# The fine art of chromatin folding: Revealing the path of DNA inside mitotic chromosomes

**DOI:** 10.1016/j.xgen.2025.100921

**Published:** 2025-06-11

**Authors:** Marina Vitoria Gomes, Christian H. Haering

**Affiliations:** 1 Chair of Biochemistry and Cell Biology, Theodor-Boveri-Institute of Biosciences, Biocenter of the Julius-Maximilians-Universität Würzburg, Würzburg, Germany

## Abstract

How chromatin fibers fold into rod-shaped mitotic chromosomes has long been a central question in genome biology. Two new studies now reconstruct the path of DNA in human chromosomes at nanoscale resolution and reveal how loop-extruding molecular machines fold chromatin into compact, rod-shaped structures at the onset of mitosis. Using microscopy- or sequencing-based approaches, respectively, both studies converge on similar models of chromosome organization yet differ in key mechanistic details. The findings further challenge textbook hypotheses of higher-order chromatin structures and reignite the quest for a detailed understanding of genome architecture inside living cells.

## Main text

At the end of the 19th century, German cytologist Theodor Boveri concluded that chromosomes, seen as “compact thread pieces” during cell division, exist as individual entities both before and after mitosis; they simply become visible during mitosis because they adopt a highly organized form.^1^ Since then, generations of cell biologists have sought to understand how loosely folded interphase chromatin fibers re-organize into rod-shaped mitotic chromosomes.

Revealing the precise architecture of mitotic chromosomes has been a major challenge, largely due to the chromosomes’ dynamic, short-lived nature and the dense packing of chromatin into a cylindrical space. In the past decade, breakthroughs in genome-wide chromatin contact mapping using high-throughput DNA sequencing (Hi-C) have provided molecular-resolution insights into mitotic chromosome folding.^2^ *In silico* polymer models replicating these interaction maps suggested that mitotic chromosomes are built from arrays of consecutive loops arranged around a central flexible core of protein complexes—named condensins—connecting the loop bases.

However, obtaining direct experimental proof for the DNA loops predicted by Hi-C-based models proved difficult. DNA loops up to ∼100 kilobase pairs (kbp) in length—matching model predictions—had been observed decades earlier in electron micrographs of histone-depleted mitotic chromosomes isolated from human cells.^3^ More recent electron tomograms resolved the positions and orientations of nucleosomes and most of the linker DNA between them in partially decondensed mitotic chromosomes.^4^ However, due to limited tilt-section volumes, the chromatin fiber path could be followed over only a few kilobase pairs—much shorter than the proposed loop lengths.

In their latest *Cell* article,^5^ Beckwith, Brunner, and colleagues mapped the path of DNA along entire chromosomes in hundreds of intact human mitotic cells. They achieved this using “LoopTrace,” a modified DNA fluorescence *in situ* hybridization (FISH) technique that, unlike earlier versions, avoids heat or chemical denaturation to produce single-stranded DNA for probe hybridization.^6^ Instead, one DNA strand is labeled with bromine nucleotide analogs during replication and then selectively digested following chemical fixation and UV-induced nicking, preserving genome structure in a near-native state.

Reconstructing the DNA path of two entire chromosomes at 1-megabase pair (Mbp) resolution enabled visualization of cell-cycle-dependent global changes in genome architecture in HeLa cells—from diffuse interphase territories to highly compacted metaphase chromosomes. Tracing of selected chromosomal subregions at higher resolution, down to 12 kbp, revealed a striking reorganization of chromatin fibers as chromosomes adopted rod-like shapes. Regularly spaced clusters of small loops—presumably corresponding to topologically associating domains (TADs)—dissolved, while larger, backfolded DNA loops became more prominent. These expanded to several hundred kilobase pairs during the transition from prophase to prometaphase, followed by the emergence of nested loops as lateral compaction of the chromosome rods increased. Supporting the prevailing model that condensin I and II complexes are responsible for actively extruding these loops, degron-mediated depletion of their shared SMC4 subunit led to loss of both large loops and nested subloops, along with a reduction in chromosome compaction.

In a complementary study recently published in *Science*,^7^ Samejima, Gibcus, and colleagues explored how the two condensin complexes, along with the related cohesin complex, contribute to mitotic chromosome folding. Using degron-mediated depletion of subunits specific to one or multiple complexes in chicken DT40 cells arrested prior to mitotic entry, the researchers tracked changes in chromosome architecture via Hi-C after synchronous release of the cells into mitosis. Polymer-based modeling of the Hi-C data revealed that depletion of the shared condensin I and II subunit SMC2 prevented the dissolution of the small, cohesin-mediated interphase loops—implying that condensin-mediated loop extrusion displaces cohesin upon encounter. These results align with the LoopTrace findings,^5^ which similarly showed a shift from small, localized loops to larger, stochastic loops during mitotic chromosome condensation.

**Figure 1:**
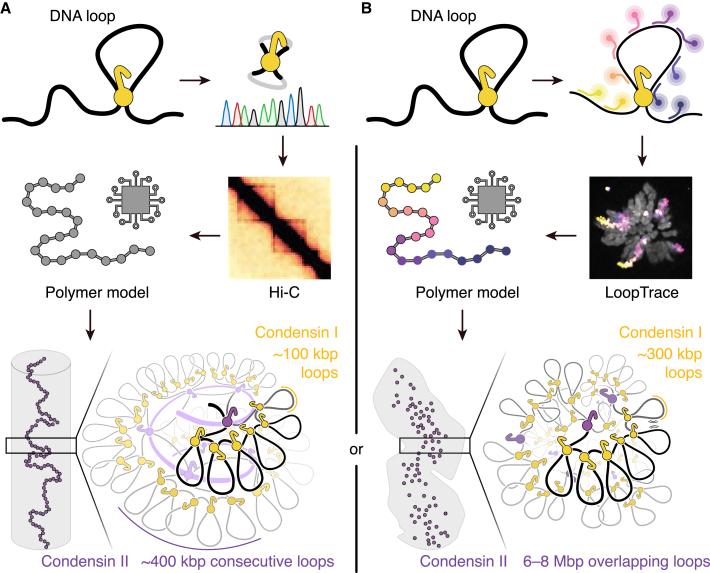
Schematic models of mitotic chromosome organization via condensin-mediated chromatin loops (A) Polymer simulations calibrated to Hi-C interaction frequency data suggest a helical arrangement of condensin II complexes (purple) positioned at the bases of consecutive chromatin loops approximately 400 kbp in length. Each of these loops is further subdivided into smaller, ∼100-kbp loops by condensin I (yellow). The axial positioning of condensin II complexes requires that they cannot pass one another, leading to a regular helical structure. For clarity, fewer loops are shown per helical turn than for the predicted DNA length (∼17 Mbp per turn). (B) Polymer simulations based on DNA FISH microscopy (LoopTrace) indicate that loop extrusion combined with DNA self-repulsion can generate straight, rod-shaped mitotic chromosomes. This model reproduces the observed central localization of condensin II and the peripheral distribution of condensin I. Here, condensin II complexes are permitted to pass one another, forming overlapping loops averaging 6–8 Mbp, which are further subdivided by condensin I into smaller, nested loops. Hi-C contact maps and LoopTrace microscopy images are reproduced from Samejima et al.^7^ and Beckwith et al.,^5^ respectively.

Cohesin depletion enhanced a feature in Hi-C contact maps that had also been observed in non-depleted DT40 cells^8^: a second diagonal of contacts, spaced 3–12 Mbp from the main diagonal representing zero contact distance. This second diagonal can be explained by a regular helical arrangement of mitotic loop bases, where each loop base aligns periodically with those from adjacent turns up and down the helix. Such a pattern brings loop bases from preceding and succeeding helical turns into spatial proximity (Figure 1A). Co-depletion of condensin I with cohesin led to the emergence of third and even fourth diagonals at intervals matching multiples of the initial spacing—consistent with loop bases contacting those two or three helical turns apart in the shorter, thicker chromosomes that result from condensin I loss.

In contrast to what has been predicted by Hi-C-based models, direct visualization of the DNA path using LoopTrace did not reveal any regular higher-order organization, such as helical coiling, at the chromosomal scale.^5^ However, plotting physical distance (from tracing) against genomic distance showed a distinct “dip” at 6–8 Mbp—coinciding with the spacing of the second diagonal observed in Hi-C. This feature disappeared upon condensin depletion.

Polymer simulations based on the DNA tracing data, combined with quantitative measurements of condensin abundance and turnover^9^ and loop extrusion properties,^10^ suggested an alternative to helical organization. The best fit to experimental observations was achieved under three conditions: (1) a small number of chromosome-bound condensin II complexes (about two per megabase pair) are allowed to freely pass one another, generating large, convoluted loops of 6–8 Mbp; (2) condensin I, which binds chromosomes only after nuclear envelope breakdown, then forms smaller, nested loops within these larger structures; and (3) chromatin self-repulsion limits excessive compaction of the polymer chain. These parameters produced cylindrical bodies resembling mitotic chromosomes, with condensin II complexes concentrated near the axial core and condensin I near the periphery (Figure 1B), as previously seen using microscopy.^9^ The key role of electrostatic contributions to chromosome folding became clear after increasing electrostatic repulsion by histone deacetylase inhibition, which reduced chromatin compaction in both simulations and LoopTrace measurements without altering condensin-driven loop characteristics. Thus, loop extrusion combined with chromatin self-repulsion is sufficient to explain the observed chromosome organization, without invoking additional structural constraints such as externally enforcing a cylindrical shape.

In contrast, polymer models based on Hi-C data and calibrated with quantitative proteomics of chromosomal condensin levels^7^ suggest that condensin II forms consecutive, non-overlapping loops of approximately 400 kbp. To account for the observed localization of condensin II along the inner axis of mitotic chromosomes, the model requires that condensin complexes cannot pass one another. Remarkably, to explain the axial localization of condensin in each chromatid pair, the model at the same time predicts that condensin complexes must be able to bypass cohesin complexes holding sister chromatids together. The conclusion that two condensin II complexes stall when they encounter each other furthermore stands in tension with the observation that yeast condensin complexes can traverse each other in *in vitro* single-molecule DNA-loop-extrusion experiments.^10^

The studies by Beckwith, Brunner, and colleagues^5^ and by Samejima, Gibcus, and colleagues^7^ offer unprecedented insight into the long-elusive fine architecture of mitotic chromosomes. Despite employing fundamentally different approaches—microscopic DNA tracing versus sequencing-based Hi-C—both groups converge on strikingly similar conclusions. Both studies show that mitotic chromosome condensation begins with condensin-II-mediated loop formation, which displaces interphase genome features—such as TADs—that were maintained by cohesin. The initial lengthwise compaction is followed by lateral condensation through the formation of smaller, nested loops driven by condensin-I-mediated loop extrusion. Both models estimate comparable loop extrusion rates of a few kilobases per second, aligning with measurements from single-molecule assays of condensin on naked DNA^10^—particularly when accounting for the additional shortening achieved as DNA is wrapped into nucleosomes.

Although both experimental approaches examined mitotic chromosomes in living cells in a near-native state, cells still required substantial processing, including chemical fixation and DNA fragmentation. Advances in cryo-focused ion beam preparation for cryoelectron tomography may soon enable tracing of chromatin fibers in flash-frozen cells across the full length of mitotic DNA loops—without the need for chromosome isolation or partial decondensation.^4^ Such methods could ultimately help resolve the controversy over whether chromatin loops are arranged in a helical order or in a more random manner. Moreover, observing condensin II complexes extrude loops on chromatinized substrates may clarify whether condensins stall upon encountering each other or bypass one another to generate overlapping loops at the megabase pair scale. What is already evident from these and other recent studies is that the traditional textbook view of mitotic chromosome formation—as a hierarchical series of regular, higher-order chromatin structures starting at the 30-nm fiber—must be revised.

## Acknowledgments

Work in the Haering group is funded by Deutsche Forschungsgemeinschaft (DFG, German Research Foundation), grant HA 5853/5-1.

## Declaration of interests

The authors declare no competing interests.
